# Utilizing HIV Proviral DNA to Assess for the Presence of HIV Drug Resistance

**DOI:** 10.1093/cid/ciaf161

**Published:** 2025-04-03

**Authors:** Annemarie M Wensing, Charlotte Charpentier, Vincent Calvez, Francesca Ceccherini-Silberstein, Huldrych F Günthard, Donna M Jacobsen, Roger Paredes, Robert W Shafer, Douglas D Richman

**Affiliations:** Department of Global Health and Bio-Ethics, Julius Center for Health Sciences and Primary Care, University Medical Center Utrecht, Utrecht, The Netherlands; Ezintsha, Faculty of Health Sciences, University of the Witwatersrand, Johannesburg, South Africa; Department of Virology, Paris Cité University and Bichat-Claude Bernard Hospital, Paris, France; Department of Virology, Sorbonne Université School of Medicine, INSERM, Institut Pierre Louis d'Epidémiologie et de Santé Publique, AP-HP, Hôpitaux Universitaires Pitié Salpêtrière—Charles Foix, Laboratoire de Virologie, Paris, France; Department of Experimental Medicine, University of Rome Tor Vergata, Rome, Italy; Department of Infectious Diseases and Hospital Epidemiology, University Hospital Zurich and Institute of Medical Virology, University of Zurich, Zurich, Switzerland; International Antiviral Society–USA, San Francisco, California, USA; Department of Infectious Diseases and irsiCaixa, Hospital Universitari Germans Trias i Pujol, Badalona, Spain; Universitat Politècnica de Catalunya - BarcelonaTech, Terrassa, Spain; Department of Medicine, Stanford University School of Medicine, Stanford, California, USA; Departments of Pathology and Medicine, University of California San Diego, San Diego, California, USA

**Keywords:** proviral DNA testing, drug resistance, HIV-1, antiretroviral drugs, long-acting drug regimen

## Abstract

The improved efficacy and tolerability of newer antiretroviral drugs, as well as the introduction of long-acting regimens, have prompted more frequent therapy switches in individuals on suppressive antiretroviral therapy (ART). For these individuals, the assessment of HIV drug resistance using DNA from peripheral blood lymphocytes has become increasingly popular. However, compared with HIV RNA-based analyses, implementation of HIV DNA testing as an alternative approach in clinical care requires new documented quality-assessment procedures and clinical validation. Furthermore, the use of HIV DNA to assess drug resistance has some distinct technical and biologic challenges that are relevant to the clinical management of people with HIV. This Viewpoint article addresses the issues relevant to clinical virologists and treating physicians for the interpretation of drug-resistance testing or subtype assessment based on DNA analysis, when HIV RNA genotypic assessment is not possible.

## Teaser

HIV RNA resistance testing is important for HIV treatment-related decisions but may not be possible in some situations. Proviral DNA testing may be an alternative in clinical trials and practice in some situations, but comes with technical and biologic limitations.

Human immunodeficiency virus (HIV) RNA genotypic resistance testing is a crucial clinically validated tool to optimize antiretroviral treatment–related decisions. The International Antiviral Society–USA (IAS–USA) HIV Drug Resistance Group maintains a list of relevant mutations in HIV that impact drug susceptibility [1] and has previously recommended HIV-RNA resistance testing for individuals who are drug naive at the time of presentation and in those experiencing failure of therapy who have an HIV-RNA level above 200 copies/mL [2]. However, there are specific situations in which a resistance test may be warranted when HIV-RNA testing is not possible. Such situations could include repeated failure of RNA amplification at lower levels of viremia or a switch to a new antiretroviral regimen in the setting of viral suppression. These may include a switch to a new regimen in the absence of a clearly documented treatment history, or insufficient access to historical resistance test results performed at baseline, or at earlier times of failure, or a switch of therapy for which information is needed that is not documented in a previous resistance report. Some of these situations may be overcome by performing HIV-RNA resistance testing on stored plasma samples; however, consistent storage of past samples is expensive and often unavailable.

Recently, there has been an increased use of proviral DNA as an alternative source for HIV resistance testing in clinical trials and in routine practice [3, 4]. Although the rationale for the potential use of resistance testing using viral DNA isolated from infected cells in the situations described above seems reasonable, several technical and biologic challenges associated with this approach need to be appreciated.

## TECHNICAL ASPECTS OF HIV DNA RESISTANCE TESTING

To perform sequencing of proviral HIV DNA variants, DNA can be extracted directly from a whole-blood sample or from peripheral blood mononuclear cells (PBMCs) and then amplified using nested polymerase chain reaction (PCR) [5]. The amplified DNA is sequenced using dideoxy-terminator Sanger sequencing or next-generation sequencing (NGS). Sanger sequencing reads individual DNA fragments, but NGS allows for massively parallel sequencing of DNA fragments, enabling higher throughput and greater sensitivity. The number of viral genomes analyzed in the procedure depends on the PBMC proviral load, the number of PBMCs from which DNA is extracted, and the efficiency of DNA extraction and PCR [5]. Amplification has been reported to be successful even in individuals with low CD4+ cell counts, likely because these individuals tend to have higher proviral DNA loads [6].

There is more random variation in the detection of drug-resistance mutations (DRMs) within a single sample when analyzing PBMCs than when analyzing plasma. This is because DRMs in PBMCs are more likely to be mixed with ancestral virus populations [5, 7]. Indeed, the mean reproducibility at detecting DRMs on repeat assays was approximately 80% in 2 different studies [8, 9]. Sampling bias is also likely to affect reproducibility when variants are present in low proportions within the proviral DNA, because fewer HIV sequences are available for interrogation. One approach to mitigate this limitation has been to perform triplicate-nested PCR [10].

In several countries, academic laboratories offer DNA resistance testing as a technically validated procedure [6]. Sanger sequencing can detect the presence of mutations at levels of approximately 20% in the viral population [11, 12]. In contrast, the proportion at which variants can be detected by NGS depends on the threshold selected by the laboratory, which usually ranges between 1% and 10%. One commercially available, technically validated DNA resistance assay using NGS has a mutation detection threshold of approximately 3% to 10%, with the lower threshold applied to mutations in sequences that are not consistent with APOBEC (apolipoprotein B mRNA editing enzyme) alterations [13], as described below.

Regardless of the assay used, the interpretation of DRMs in proviral DNA requires an approach that considers the near-universal presence of APOBEC-mediated G-to-A hypermutation at some level in PBMC HIV DNA [14, 15]. APOBEC 3F and 3G are host enzymes that cripple viral genomes by indiscriminately mutating the dinucleotides GG to AG and GA to AA, respectively. In most cases, sequences that contain APOBEC3-mediated G-to-A hypermutation also have stop codons resulting from G-to-A changes at tryptophan amino acids or changes from aspartate to asparagine at 1 or more active site positions. The DRMs resulting from G-to-A hypermutation (rather than from drug selection pressure) are likely to be present in replication-incompetent viruses that also contain stop codons and other crippling mutations.

Eighteen DRMs arise within an APOBEC3-mediated dinucleotide context (Table 1) [16]. For NGS, 2 approaches can be applied to account for APOBEC3-mediated DRMs: (1) excluding sequence reads that are hypermutated or (2) determining the proportion of sequences that are hypermutated and then reporting only those APOBEC3-context DRMs that occur above this proportion. Of note, studies using long-read NGS showed that stop codons and G-to-A hypermutation are mostly present on the same read [17]. It is essential that laboratory drug-resistance reports confirm that APOBEC3-mediated mutations have been purged from DNA resistance results when using NGS sequencing.

For Sanger sequencing it can be more challenging to discriminate whether resistance mutations are present in replication-competent variants or in APOBEC-mediated defective virus [18]. DNA resistance reports based on Sanger sequencing should acknowledge this limitation and indicate which mutations may be mediated by APOBEC. In addition, it is crucial that publications and presentations about studies using HIV DNA drug-resistance testing indicate that the effects of APOBEC editing have been accounted for in the results.

## POTENTIAL INDICATIONS, INTERPRETATION, AND LIMITATIONS OF HIV DNA RESISTANCE TESTS

### Potential Indications

There are several situations in which HIV DNA resistance analysis may have added value (see Text box), as follows:

1. Repeated failure of RNA amplification at baseline or time of virologic failure, especially at lower levels of viremia when amplification of HIV-RNA may fail.
2. A switch to a new regimen in individuals who are virally suppressed in the absence of a clearly documented treatment history, or insufficient access to historic resistance tests performed at baseline or during previous failure:
  - When a switch is considered to a regimen with a lower genetic barrier to resistance than the current suppressive regimen.
  - When recycling a drug class that was previously used at times of insufficient or unknown levels of viral suppression.
  - When using a drug in the nonnucleoside analogue reverse transcriptase inhibitor (NNRTI) class if an NNRTI-based regimen was previously interrupted without immediate switch to a new regimen since NNRTI resistance may have been selected after interruption due to prolonged presence of insufficient drug levels [19, 20].
  - When the viral subtype is not recorded in previous reports. This information may be useful when selecting antiretroviral drugs in certain geographic regions. Also, an earlier reported subtype may not be adequately classified based on current knowledge. This is particularly relevant for subtype A6, which has been often misclassified as subtype A1. Subtype A6 has been recently identified as 1 of the risk factors for virologic failure of the injectable long-acting combination of the NNRTI rilpivirine and the integrase strand transfer inhibitor (InSTI) cabotegravir [21].
  - When insight regarding resistance-related polymorphisms is needed. Circulating wild-type viral polymorphisms may diminish antiviral activity or lower the genetic barrier to clinically relevant resistance to certain drugs. Particularly the reverse transcriptase (RT) E138A polymorphism related to rilpivirine and the integrase E157Q polymorphism related to low-level resistance to the first-generation InSTIs raltegravir and elvitegravir. Each of these mutations occurs in 1% to 6% of isolates from antiretroviral therapy (ART)–naive persons depending on subtype [22–24].
  - When co-receptor tropism is required for a switch to the CCR5-receptor inhibitor maraviroc. In general, a high concordance of co-receptor tropism assignment based on RNA and DNA has been reported in sample sets with predominantly CCR5-tropic viruses in the plasma. However, CXCR4-tropic viruses may be more often detected in DNA than in RNA [25, 26].
3. The initiation of therapy in individuals not on ART, since in the absence of selective pressure, resistant variants may be archived in the DNA but replaced in the plasma by more replication-competent variants with less or no resistance.
  - When, in individuals who are previously ART exposed, ART is reinitiated after discontinuation for several weeks without assessment of resistance at time of earlier (presumed) therapy failure and without access to a stored plasma sample at the time of failure [27, 28].
  - When individuals who are chronically infected and therapy-naive initiate ART while there is a high likelihood of initial infection with drug-resistant HIV. In cases of infection with resistant virus, no wild-type virus is archived in the DNA. The chance of detection of resistance mutations in the plasma differs depending on the fitness costs they induce. Mutations may be still detectable in DNA once they have cleared from plasma [13, 29–31].

**Text box:** Potential Usefulness of DNA Resistance Testing

HIV DNA testing may be useful when RNA resistance testing results cannot be generated due to inability to perform RNA amplification.
HIV DNA testing could provide supplementary information on previously selected resistance in individuals who are suppressed with antiretroviral therapy when there is an incomplete therapeutic history, absence of sequence results, or missing information on earlier reports about subtype or coreceptor tropism.
HIV DNA testing could provide supplementary information on baseline resistance either due to resistance-related polymorphisms or transmitted resistance.

Abbreviation: HIV, human immunodeficiency virus.

**Table 1.** Interpretation of Genotypic Resistance Tests From Proviral DNA

Indication	Interpretation	Comments
Determining HIV-1 subtype	Useful	The HIV subtype can be reliably determined from proviral HIV DNA
Determining HIV co-receptor tropism	Potentially useful	X4-tropic virus is more often detected in proviral HIV DNA
Identifying DRMs		
DRMs not related to APOBEC3 editing	Potentially useful	These mutations may be present in replication-competent virus and should be taken into account
DRMs that could arise from APOBEC3 editing • PIs (D30N, M46I, G48S, G73S) • nRTIs (D67N and M184I) • NNRTIs (E138K, G190E, G190S, and M230I) • InSTIs (G118R, E138K, G140R, G140S, G163K, G163R, D232N, and R263K)	Uncertain	These mutations are often present in replication-incompetent HIV that have other APOBEC3-derived mutations, such as stop codons^a^
		Ensure that the sequencing laboratory has accounted for APOBEC3-induced mutations
		Check the mutation detection threshold if NGS has been applied: lower thresholds (eg, 2%) provide less reliable information than higher ones (eg, >10%)
Absence of DRMs	Uncertain	Proviral DNA testing has lower sensitivity and may miss relevant DRMs^b,c^

Abbreviations: APOBEC3, apolipoprotein B mRNA editing enzyme, catalytic subunit 3; DRM, drug-resistance mutation; HIV, human immunodeficiency virus; InSTI, integrase strand transfer inhibitor; NGS, next-generation sequencing; NNRTI, nonnucleoside analogue reverse transcriptase inhibitor; nRTI, nucleoside or nucleotide analogue reverse transcriptase inhibitor; PBMC, peripheral blood mononuclear cell; PCR, polymerase chain reaction; PI, protease inhibitor.

Technical challenges of proviral DNA genotyping affecting clinical interpretation:

^a^APOBEC3 editing of integrated HIV proviruses may introduce mutations associated with drug resistance alongside other mutations or stop codons, which make the integrated HIV provirus replication incompetent.

^b^The number of viral genomes able to be sampled depends on the PBMC proviral copy number, the number of PBMCs from which DNA is extracted, and the efficiencies of DNA extraction and PCR amplification.

^c^There is greater intra-sample stochastic variation in the detection of DRMs in PBMCs than in plasma, because DRMs in PBMCs are more likely to coexist with ancestral wild-type virus populations. In addition, plasma is a homogeneous liquid source, but the number of HIV-infected CD4+ cells can vary in the PBMCs according to the immunologic status of the individual.

### Interpretation and Limitations

Various randomized clinical studies have shown the added value of HIV RNA resistance testing to guide treatment decisions, but no such studies have been performed for HIV DNA resistance testing [32–34]. As HIV DNA resistance tests are now commercially available and used in clinical trials to assess resistance, it is important that clinicians are aware of the limitations and uncertainties regarding the use of proviral DNA to detect drug resistance for guiding treatment decisions (Table 1). HIV DNA resistance test results thus need to be interpreted with caution. HIV DNA resistance testing should only be considered when plasma HIV RNA testing is not technically feasible. When making treatment decisions, one must take into account the treatment history, the history of previous treatment failures, and the alternative treatment options available for that particular individual. It is uncertain whether HIV DNA resistance testing information will lead to better treatment choices than clinical judgment based on these considerations.

On the one hand, clinicians must be aware that drug-resistant variants may be missed by proviral DNA testing. At the time of therapy failure, RNA resistance testing is preferred since resistance mutations are detected in the plasma earlier than in DNA [35, 36]. Also, in ART-suppressed individuals, the negative-predictive value of HIV DNA resistance tests is lower than the evaluation of cumulative historical RNA-based resistance tests performed at the time of failure [37, 38]. This is likely based on the fact that decline in archived resistant viruses in the viral reservoir increases with the duration of virologic suppression, once an effective ART regimen is initiated after virologic failure [39, 40]. The kinetics of decay depend on the DRMs and several other factors, such as the duration and level of viral replication at the time of virologic failure [41]. In addition, only a subset of as much as 2 to 3 million PBMCs is assessed with HIV DNA resistance tests, representing a very small fraction of the viral reservoir. In addition, these cells may not be informative of the viral reservoir that is present in lymph nodes or other anatomic compartments. If Sanger technology is used for sequencing, only viral variants representing more than 20% of all viral quasi-species may be detected and minority resistant variants may be overlooked. This limitation is likely more relevant considering the overall decreased sensitivity of HIV DNA testing compared with RNA testing. Selection of therapy based on the absence of DRMs in an HIV DNA test may therefore be risky, especially when switching to a regimen with a lower genetic barrier to resistance.

On the other hand, DNA resistance testing may overestimate resistance. The viral reservoir is constituted primarily of defective proviruses, mainly due to large internal deletions and not just due to APOBEC-induced mutations [14]. Some DRMs detected in proviral DNA may occur in nonviable viruses and thus unlikely to cause virologic failure. Avoiding a certain treatment based on the detection of DRMs with an HIV DNA test may be too restrictive and disqualify beneficial therapeutic options.

## CONCLUSIONS

The use of HIV DNA resistance tests can lead to an underestimation of DRMs due to technical limitations of the methodology and to a low negative-predictive value, as well as possible overestimation of resistance mutations due to the presence of APOBEC3-induced defective proviruses. HIV drug-resistance testing using DNA may have added value for the indications described in this article. Given the limitations and technical challenges, vigorous quality control is warranted. It is important that resistance reports clearly indicate when resistance results are based on DNA. Implementation of antiretroviral drug-resistance testing using viral DNA in clinical practice with increased confidence will require additional rigorous clinical investigation.

## Notes

***Author Contributions*.** Concept and design: A. M. W. and D. D. R. Drafting of the manuscript: all authors. Critical review of the manuscript for important intellectual content: all authors. All authors have approved the final manuscript to be published.

***Acknowledgments*.** The IAS–USA HIV Drug Resistance Group is an independent, volunteer panel of experts charged with delivering accurate, unbiased, and evidence-based information on drug resistance–associated mutations for HIV virologists and clinicians. The authors thank Ami Peltier for editorial assistance with the work. This work is dedicated to the memory of our friend and colleague, Victoria A. Johnson, MD.

***Financial support.*** This work was supported by the International Antiviral Society (IAS)–USA. No commercial or government support was used.
